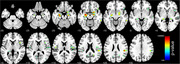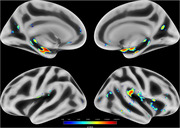# Increased cortical mean diffusivity in cognitively unimpaired individuals with elevated plasma ptau217

**DOI:** 10.1002/alz70862_110080

**Published:** 2025-12-23

**Authors:** Linda Zhang, Jesús Silva‐Rodríguez, Eva Alfayate, Sonia Wagner, Pascual Sanchez‐Juan, Michel J. Grothe

**Affiliations:** ^1^ CIEN Foundation, Reina Sofia Alzheimer Center, ISCIII, Madrid, Madrid Spain

## Abstract

**Background:**

Plasma ptau217 has recently emerged as a reliable biomarker for early Alzheimer’s disease (AD) pathology. Additionally, previous works have suggested that indices of cortical mean diffusivity (MD) derived from diffusion‐weighted imaging are thought to reflect early microstructural changes preceding detectable structural atrophy in neurodegenerative diseases. While ptau217 has been shown to correlate with cortical atrophy in cognitively normal individuals, its relationship with cortical MD remains unstudied.

**Methods:**

1029 cognitively unimpaired (CU) elderly participants (mean age: 74.9, range: 69‐87; 65% female) with 3T MRI and plasma ptau217 measures were selected for baseline cross‐sectional analyses from the Vallecas Project cohort, a single‐centre 12‐year longitudinal study with annual follow‐ups. Plasma ptau217 levels were measured on the fully automated LUMIPULSE platform, and individuals were classified as either ptau217+ (*n* = 174, mean age: 76.0) or ptau217‐ (*n* = 855, mean age: 74.7) based on a pre‐established threshold of 0.247 pg/mL. Diffusion‐weighted images were preprocessed using an in‐house pipeline to correct for eddy currents and image distortions due to magnetic field inhomogeneity, and diffusion tensors were fitted using FSL. A normalised grey matter image was used to mask normalised MD images to obtain cortical grey matter MD maps, which were subsequently analysed in a voxel‐wise GLM using SPM12, controlling for age and sex.

**Results:**

At baseline, there was no difference between groups in sex distribution, although individuals with elevated ptau217 levels were significantly older (*p* = 0.0001). After controlling for sex and age, the ptau217+ group showed significantly higher cortical MD in bilateral medial temporal (amygdala and hippocampus) and insular regions (insular and opercular cortices) compared to ptau217‐ (*p* <0.05, FDR‐corrected; Figure 1).

**Conclusions:**

In our study of a large‐scale, well‐characterised cognitively unimpaired population with available diffusion weighted imaging, changes in cortical MD can already be observed in those with elevated plasma ptau217 in regions commonly associated with AD neuropathology. Further analysis on longitudinal MD changes in the same cohort are ongoing.